# A new species of *Cheironitis* van Lansberge, 1875 from Jordan (Coleoptera, Scarabaeidae, Onitini)

**DOI:** 10.3897/BDJ.9.e69763

**Published:** 2021-10-28

**Authors:** Paul Coppo, Olivier Montreuil

**Affiliations:** 1 AP-HP, Paris, France AP-HP Paris France; 2 MNHN, Paris, France MNHN Paris France

**Keywords:** dung beetle, Jordan, Middle East, new species

## Abstract

**Background:**

The genus *Cheironitis* van Lansberge, 1875, currently contains 23 species from the Old World. During a survey for dung beetles in Jordan, specimens of an undescribed species were collected at the historical site of Petra.

**New information:**

A new species of *Cheironitis* (*C.petraensis* sp. n.) is described from the historical site of Petra, Jordan, illustrated and compared with its most closely related species. This new species is reminiscent of the African species of *Cheironitis* living in savannahs and could represent a relictual species of the mid-Holocene climatic optimum.

## Introduction

The genus *Cheironitis* van Lansberge, 1875, currently contains 23 species from the Old World, including 13 in Palaearctic and 10 in Afrotropical Regions (Anonymous 2021).

While surveying dung beetles in Jordan in July 2012, the first author collected specimens of an undescribed species of dung beetle from horse dung at the historical site of Petra. This species belongs to a group of fulvous-coloured species, characterised by rows of smooth and shiny black tubercles on the elytra, including *C.asbenicus* Gillet, 1909, from southern Sahara, *C.scabrosus* (Fabricius, 1776), from south-eastern Africa, *C.muelleri* Janssens, 1943, from eastern Africa and *C.socotranus* Gahan, 1909, from Socotra Island (Balthasar 1963, Balthasar 1963, Bezdek 2016, Janssens 1937, Janssens 1943). This species is described here as new and compared with its most closely related species.

## Materials and methods

Dry specimens and dissected structures were observed using a Bresser Advance ICD10-160X microscope. Illustrations were made using a Canon EOS 6D Mark II, coupled with a Canon EF 100mm f/2.8L Macro USM and a Macro Ring Lite MR-14EX. Images were stacked using Helicon Focus software.

## Taxon treatments

### Cheironitis petraensis

Coppo sp. n.

2FF18401-02DF-5B50-8EAB-33B49FC47C5F

63675D84-2605-4659-8030-75E46ADFC550

#### Materials

- **Type status:** Holotype. **Occurrence:** sex: male; lifeStage: adult; **Taxon:** scientificName: Cheironitispetraensis; order: Coleoptera; family: Scarabaeidae; genus: Cheironitis; specificEpithet: petraensis; taxonRank: species; scientificNameAuthorship: Coppo; **Location:** country: Jourdan; locality: Petra historical site, Street of Facades; verbatimLocality: 30°19'43.7"N, 35°26'43.7"E; verbatimElevation: 910 m; locationRemarks: horse dung on dirt road; **Identification:** identifiedBy: Paul Coppo & Olivier Montreuil; dateIdentified: 2021-05-23; **Event:** eventDate: 2012-7-18; **Record Level:** collectionID: Paul Coppo collection, Paris, France; basisOfRecord: PreservedSpecimen
- **Type status:** Paratype. **Occurrence:** sex: male; lifeStage: adult; **Taxon:** scientificName: Cheironitispetraensis; order: Coleoptera; family: Scarabaeidae; genus: Cheironitis; specificEpithet: petraensis; taxonRank: species; scientificNameAuthorship: Coppo; **Location:** country: Jourdan; locality: Petra historical site, Street of Facades; verbatimLocality: 30°19'43.7"N, 35°26'43.7"E; verbatimElevation: 910 m; locationRemarks: horse dung on dirt road; **Identification:** identifiedBy: Paul Coppo & Olivier Montreuil; dateIdentified: 2021-05-23; **Event:** eventDate: 2012-7-18; **Record Level:** collectionID: Paul Coppo collection, Paris, France; basisOfRecord: PreservedSpecimen
- **Type status:** Paratype. **Occurrence:** sex: male; lifeStage: adult; **Taxon:** scientificName: Cheironitispetraensis; order: Coleoptera; family: Scarabaeidae; genus: Cheironitis; specificEpithet: petraensis; taxonRank: species; scientificNameAuthorship: Coppo; **Location:** country: Jourdan; locality: Petra historical site, Street of Facades; verbatimLocality: 30°19'43.7"N, 35°26'43.7"E; verbatimElevation: 910 m; locationRemarks: horse dung on dirt road; **Identification:** identifiedBy: Paul Coppo & Olivier Montreuil; dateIdentified: 2021-05-23; **Event:** eventDate: 2012-7-18; **Record Level:** collectionID: Paul Coppo collection, Paris, France; basisOfRecord: PreservedSpecimen
- **Type status:** Paratype. **Occurrence:** sex: female; lifeStage: adult; **Taxon:** scientificName: Cheironitispetraensis; order: Coleoptera; family: Scarabaeidae; genus: Cheironitis; specificEpithet: petraensis; taxonRank: species; scientificNameAuthorship: Coppo; **Location:** country: Jourdan; locality: Petra historical site, Street of Facades; verbatimLocality: 30°19'43.7"N, 35°26'43.7"E; verbatimElevation: 910 m; locationRemarks: horse dung on dirt road; **Identification:** identifiedBy: Paul Coppo & Olivier Montreuil; dateIdentified: 2021-05-23; **Event:** eventDate: 2012-7-18; **Record Level:** collectionID: Paul Coppo collection, Paris, France; basisOfRecord: PreservedSpecimen
- **Type status:** Paratype. **Occurrence:** sex: female; lifeStage: adult; **Taxon:** scientificName: Cheironitispetraensis; order: Coleoptera; family: Scarabaeidae; genus: Cheironitis; specificEpithet: petraensis; taxonRank: species; scientificNameAuthorship: Coppo; **Location:** country: Jourdan; locality: Petra historical site, Street of Facades; verbatimLocality: 30°19'43.7"N, 35°26'43.7"E; verbatimElevation: 910 m; locationRemarks: horse dung on dirt road; **Identification:** identifiedBy: Paul Coppo & Olivier Montreuil; dateIdentified: 2021-05-23; **Event:** eventDate: 2012-7-18; **Record Level:** collectionID: Paul Coppo collection, Paris, France; basisOfRecord: PreservedSpecimen
- **Type status:** Paratype. **Occurrence:** sex: female; lifeStage: adult; **Taxon:** scientificName: Cheironitispetraensis; order: Coleoptera; family: Scarabaeidae; genus: Cheironitis; specificEpithet: petraensis; taxonRank: species; scientificNameAuthorship: Coppo; **Location:** country: Jourdan; locality: Petra historical site, Street of Facades; verbatimLocality: 30°19'43.7"N, 35°26'43.7"E; verbatimElevation: 910 m; locationRemarks: horse dung on dirt road; **Identification:** identifiedBy: Paul Coppo & Olivier Montreuil; dateIdentified: 2021-05-23; **Event:** eventDate: 2012-7-18; **Record Level:** collectionID: Paul Coppo collection, Paris, France; basisOfRecord: PreservedSpecimen

#### Description

**Figure 1.**
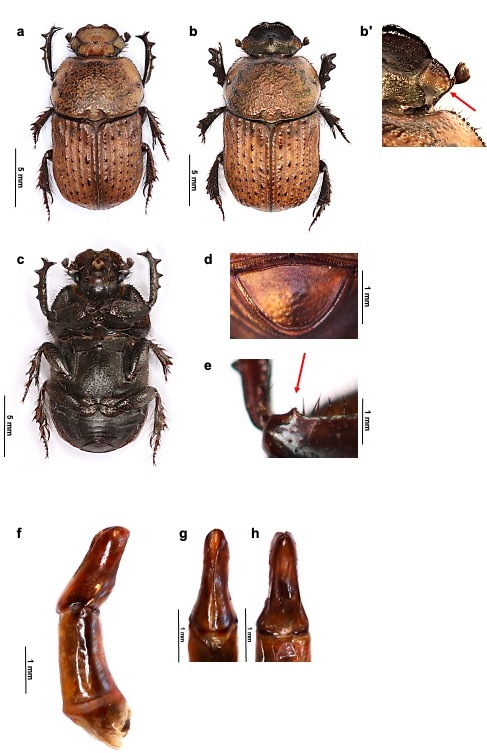
*Cheironitispetraensis* sp. n. **a** habitus male holotype dorsal view **b** habitus female paratype, dorsal view **b**' detail of female yellow-ochre gena (arrow) **c** male holotype, ventral view **d** details of pygidium **e** right fore leg, slightly oblique ventral view; arrow shows sub-apical tooth on upper edge of profemur **f** aedeagus, left lateral view **g** parameres, dorsal view **h** parameres, ventral view.

Holotype ♂ (Fig. 1a). **Overall aspect.** Length 16 mm. Body elongate and parallel, with slight metallic sheen prevailing on pronotum. **Head**. Finely granulose, yellow-ochre, bordered with dark brown. Clypeus emarginated at apex with a dark brown clypeal carina. Frontal carina dark brown, slightly curved and interrupted medially by a tubercle. Vertex sinuated backwards. Antenna brown with dark brown bristles, club black. **Pronotum.** Yellow-ochre with irregular black callosities approximately set symetrically and sparing lateral borders; disc with interspersed, coarse and deep punctures, becoming less deep and more scattered on posterior angles, each point with a granule. Basal impressions deep and curved. **Scutellar shield.** Triangulate, acute, smooth. **Elytra.** Elongate, raised basally; the dorsal surface sinuated laterally past the humeral umbone, striations finely punctuated, elytral intervals almost imperceptibly punctuated, yellow-ochre matte, each displaying an irregular row of black shiny tubercles. Lateral carina weak on first half and vanishing thereafter. **Underside** (Fig. 1c). Dark brown with lighter sides on fresh specimens, with dark brown bristles. Prosternal protrusion straight and slightly forked apically. Mesoventrite with short tubercle. Metaventrite granulate and pubescent laterally, with a slight medial groove along mid-line. **Pygidium.** Shagreened, aciculate, with brown patches (Fig. 1d). **Legs.** Outer surface of femora and tibiae yellow ochre; inner surface dark brown. Profemora display a short tooth at the antero-inferior edge proximally (Fig. 1e). Protibiae straight and not curved inwardly before middle. Distance between basal and second tooth shorter than between remaining teeth; anteroventral edge with four teeth, the proximal and distal ones short, the median one longer. Metafemora elongate, posterior edge with strong outwardly curved tooth medially. Mesofemora, meso- and metatibiae normal. **Aedeagus.** Phallobase as long as parameres. Parameres notched at proximal third; ventral border straight to the distal edge (Fig. 1f - h).

**Variation.** Measurements (3 ♂, 3 ♀). Length: male 13.0 - 16.0 mm (15.0 ± 2.1), female 15.0 - 18.0 mm (16.3 ± 1.2). **Female** (Fig. 1b). Larger. Head black, except for a patch of yellow-ochre on posterior surface of gena (Fig 1 - b'); clypeal surface coarsely granulose. Tubercle of the frontal carina stout. Metaventral groove less pronounced. Pygidium punctuated. Pronotum and elytra as in the male. Legs black. Femora unarmed. Protibiae unmodified.

**Etymology.** This new species is named after the place where it was collected, i.e. Petra historical site.

**Distribution.** To date, this species is only known from Petra historical site, Jordan.

## Analysis

*Cheironitispetraensis* sp. n. shows a stout outwardly curved long tooth medially on the posterior edge of hind femur, which is neither observed in *C.scabrosus*, *C.asbenicus*, *C.muelleri* nor in *C.socotranus*. The new species is close to *C.socotranus* by the distinct pronotal punctation of the elytral disc, the mesotibia without strong protrusion on the outer edge and the protibia straight on the basal two-thirds. Conversely, *C.socotranus* differs from the new species by several characters: underside of protibia with a row of numerous small teeth, with a much longer tooth in the middle; basal tooth of the outer edge of protibiae distinctly separated from the others; strong metaventral granulation; median coxae with a bifurcated, lamellar protrusion; the frontal tubercle of the male head distinctly behind the frontal carina; tibia with greenish reflection. *Cheironitisscabrosus, C.asbenicus* and *C.muelleri* differ from the new species by the indistinct pronotal punctures with coalescent points; by protibiae curved from the mid-length; by the presence of a strong protrusion on the inner border of protibiae and a strong and long protrusion on the outer edge of the mesotibiae. As with *C.socotranus*, they show also a different armature on underside of the protibiae, including a strong tooth, except in *C.asbenicus* where there is no tooth. *Cheironitispetraensis, C.asbenicus* and *C.muelleri* have the two basal external teeth of the protibiae slightly separated from the two apical teeth, the four teeth being equally separated in *C.scabrosus*.

Table 1 is given to separate these species.

## Discussion

Specimens of *C.petraensis* sp. n. were collected from horse dung, without other associated species. To date, this new species seems localised to the hill country of Jordan. July corresponds to a very dry period in this area, which confirms that *C.petraensis* sp. n. is a dry season active species, as are all other species of the genus. It is reminiscent of African species of *Cheironitis* living in savannahs, suggesting that *C.petraensis* sp. n. could represent a relictual species of the mid-Holocene climatic optimum (Anonymous 2012).

## Supplementary Material

XML Treatment for Cheironitis petraensis

## Figures and Tables

**Table 1.** Comparison of main morphological characters between *Cheironitispetraensis* sp. n. and its most morphologically and geographically closely related species.

	*C.scabrosus*	*C.socotranus*	*C.asbenicus*	*C.muelleri*	*C.petraensis* sp. n.
Frontal tubercle of male head	Slightly behind the frontal carina	Distinctly behind the frontal carina	Slightly behind the frontal carina	Slightly behind the frontal carina	Interrupting the frontal carina medially
Pronotal disc punctures	Indistinct, coalescent points	Distinct, only few punctures are coalescent	Indistinct, coalescent points	Indistinct, coalescent points	Distinct
Disc of metaventrite	With large, densely distributed punctures, giving rough aspect	With very strong and large granules	With large, densely distributed punctures, giving rough aspect	With large, densely distributed punctures, giving rough aspect	With small granules
External teeth of male protibiae	Equally distributed	Basal tooth of outer edge distinctly separated from the others	The two basal teeth distinctly separated from the two apical teeth	The two basal teeth slightly separated from the two apical teeth	The two basal teeth slightly separated from the two apical teeth
Shape of male protibiae	Curved from the mid-length	Straight in their basal two-thirds	Curved from the mid-length	Curved from the mid-length	Straight in their basal two-thirds
Protrusion on the inner border of male protibiae	Present, spine-shaped, directed inwards	Absent	Present, T-shaped	Present, spine-shaped, directed forwards	Absent
Underside of male protibiae	Row of numerous small teeth, with a much longer tooth at the basal third	Row of numerous small teeth, with a much longer tooth in the middle	No teeth, but small crenulations	A long tooth in the middle and a sub-apical strong protrusion	Four teeth, the most proximal and distal ones small, the two others longer
Basal protrusion on outer edge of male mesotibiae	Strong, short	Small	Long and thin, straight	Long and thin, rounded	Small
Posterior edge of male metafemora with a strong outwardly curved tooth medially	Absent	Absent	Absent	Absent	Present
Distribution	Southern Africa	Socotra Island	Southern Sahara	Eastern Africa	Jordan
